# Awe experiences, the sublime, and spiritual well-being in Arctic wilderness

**DOI:** 10.3389/fpsyg.2022.973922

**Published:** 2022-08-22

**Authors:** Helga Synnevåg Løvoll, Knut-Willy Sæther

**Affiliations:** ^1^Department of Physical Education, Volda University College, Volda, Norway; ^2^Department of Religious Studies, Volda University College, Volda, Norway

**Keywords:** eudaimonia (well-being), aesthetic experiences, Arctic wilderness, reflexive thematic analysis, interdisciplinarity and interdisciplinary team dynamics

## Abstract

Experiences of awe can carry the potential for life-transforming experiences and foster awareness of nature as a lifelong value. How these experiences emerge was investigated empirically in a pristine natural environment and analyzed informed by a bottom-up perspective with an interdisciplinary understanding of environmental aesthetics and positive psychology. A group of Arctic nature guide students (*n* = 34) was followed on an 8-day advanced glacier course with additional learning topics related to the Arctic landscape and history, wildlife, and how to protect a wilderness camp from polar bear attacks. After this experience, students were invited to participate in the research project and were asked to reflect on their experiences immediately after their return to civilization. Two narratives each from 27 participants were collected, which provided 54 quotations for interpretation. Reflexive thematic analysis (RTA) surfaced three main themes: context, human response to encounters with nature, and transformation. The study of awe brings the tension between spirituality and well-being closer. The findings add empirical knowledge to the understanding of the contexts for these highly affective and complex feelings. The findings also add refined knowledge about the relationship between awe and the sublime. In transformation for human well-being, the role of self-knowledge or self-transcendence surfaced by wilderness experiences should not be underestimated.

## Introduction

Human responses to awe experiences in nature can be recognized as aesthetic experiences. In the study of Arctic wilderness experiences, this entry might help us understand the attraction and power of adventures in the wilderness. Seeking meaning in life is seen as a central element of the conceptualization of eudaimonic well-being (Steger, 2016), where to seek to find meaning in life is not only a personal question but also a spiritual one. One approach is to know one’s core self (Schlegel et al., 2016). Here, morality is seen as part of people’s self-definition, where the true self is a source of understanding people’s values. To find situations where values are made conscious is thus an important way to understand eudaimonic well-being. Studying persons spending time in nature could be a source to identify such processes.

Empirical studies give a voice to how aesthetic experiences relate to well-being, such as the value of affective states that are produced in this process (Mastandrea et al., 2019). Specifically, aesthetic experiences in nature relate to evoking wonder and awe (Graves et al., 2020; Løvoll et al., 2020). What we consider positive emotions from these experiences are strong feelings of connectedness to nature and feeling at home in nature. However, the context and the characteristics of these experiences differ, and many questions remain unanswered to understand how these experiences emerge.

Following Ryff (2021), the centrality of nature in understanding deeper meanings for what our souls need is critical for our individual and planet’s well-being. Thus, studies of spirituality within wilderness experiences are at the heart of understanding eudaimonia. There is a deep connection between spirituality and well-being, which is only rarely discussed in the well-being literature. Referring to Ryff (2013, p. 2), a fundamental question is whether spirituality is best formulated as something that constitutes part of what defines well-being or is better construed as a separate realm that influences aspects of well-being. Living a life of purpose and experiencing personal growth in the wilderness over time could be investigated as well-being and spiritual matters. We do not limit spirituality to religions or religious worldviews but understand spirituality in a broader sense. Such a broad approach might include what Ivo Jirásek (2013) describes as non-religious spirituality. For Jirásek, non-religious spirituality emphasizes spirituality within the dimension of naturalness and does not enter the realm of the sacred. For Jirásek (2013), spirituality encompasses searching for the purpose of life, being aware of the height and depths of life, unraveling ethical and aesthetic dimensions of the world, having a desire for harmony, and—finally—hoping for an experience of transcendence. In line with Jirásek, we identify spirituality as a core element of human experience and believe it is about recognizing the sense or belief that there is something greater than ourselves or as referring to experiences beyond ordinary human nature (Jirásek, 2013). Spiritual experiences expand personal boundaries and, therefore, can be related to experiences described as self-transcendent.

People who are connected to nature tend to have greater eudaimonic well-being, finding an especially strong relationship between personal growth and being deeply engaged in nature in an emotional, experiential, or cognitive way (Nisbet et al., 2011; Capaldi et al., 2015; Pritchard et al., 2020). Thus, the conceptual understanding of connection and engagement with wilderness could have many gateways. In the psychological exploration of awe experiences, introduced by conceptual thinking by Keltner and Haidt (2003), the characteristics of vastness and the need for accommodation were identified as necessary ingredients. Later, several other characteristics of this complex experience were identified, such as the six factors on the Awe Experience Scale (Yaden et al., 2019): altered time perception, self-diminishment, connectedness, perceived vastness, physical sensations, and the need for accommodation. In an extension of this scale, a factorial analysis of three measurements of awe—the modified Differential Emotions Scale (mDES), the awe sub-scale of the Dispositional Positive Emotion Scale (DPES), and the Awe Experience Scale (AWE-S)—gave support for the need for an overarching conceptual understanding of awe with three identified factors: “belonging,” “raised above affairs,” and “imagination” (Clewis et al., 2021). In this empirical investigation, awe and the sublime were highly correlated.

Based on the development of the Sublime Emotion toward Nature (SEN) questionnaire, Bethelmy and Corraliza (2019) found sublime experiences include more than awe experiences, which was the first factor, and found the other factor is “inspiring energy.” In this approach, sublime experience is the overarching term. Thus, there are dividing and overlapping contents in the understanding of the sublime.

The relationship between awe experiences and sublime experiences has further been discussed through a “conceptual geography” (Arcangeli et al., 2020). Researchers argued that sublime experiences could be a species of awe, namely, aesthetic awe. This position, which divides awe into aesthetic, religious, and political awe and identifies the sublime as a subcategory of aesthetic awe, was supported in a philosophical investigation of these concepts (Clewis, 2021). Utilizing Sandra Shapshay (2019) framework, the sublime as an experience of immediate affective arousal relates to “thin sublime” experiences, which can be interpreted as opposed to a highly intellectual aesthetic response, “thick sublime.”

The development of psychological approaches to awe experiences is promising, but inspired by further philosophical discourses of awe and the sublime (Clewis, 2021), there is a need for inductive approaches in the intersection between philosophy and psychology to understand this complex phenomenon in more depth. Recently, psychologists started developing the psychology of the sublime. Typically, how experiences in nature relate to transformative experiences are at the center of the investigation. As this investigation started relatively recently, the empiric-driven theory of understanding the relationship between “awe” and “the sublime” is only loosely captured, and there are contradicting findings in the search for overarching terms: are awe experiences a part of the sublime, or is the sublime a part of awe experiences?

Experiences of awe in nature can be understood as sublime aesthetic experiences, which have characteristics other than traditional perceptions of beauty (Graves et al., 2020). In philosophical aesthetics, nature as a context for investigating aesthetic experiences has been neglected (Sæther, 2017). However, a revised and broader approach to aesthetic experiences has been advocated (Stecker, 2005). Sublime experiences are recognized as stronger, more powerful affective feelings compared with beauty (Graves et al., 2020). These feelings are related to life satisfaction and personal growth. According to Ledley (2009, p. 248), sublime experiences cause a sense of exaltation and awe, and Kathryn A. Ledley (2009) describes these experiences as pleasurable in terms of overwhelming. We recognize sublime experiences as attractive, yet terrifying. Thus, two effects of sublime aesthetic experiences are predominant: wonder and awe (Løvoll et al., 2020). As for the sublime, wonder and awe are overly complex phenomena and can span a wide range of overlapping understandings, such as feelings, emotions, and experiences. Overall, wonder and awe are phenomena described as being deep, immersive, having different degrees of intensity, self-transcendent, and—finally—having a spiritual, transformative quality, which carry implications for human well-being. Transformation is about activating specific virtues, stimulating personal growth, and providing a deeper understanding of humans’ relationship with nature. Vasalou (2012) describes such a spiritual transformation as developing a “spiritual consciousness” rooted in humans’ physical bodies, which yields spiritual consciousness of here and now, mystery, values, and ethical practices.

According to the communication theory of emotions (Oatley, 1992), emotions are at the center of how we recognize and understand events with an integrative perspective of how we evaluate our experiences, understand our goals and plans, and develop action readiness. An exploration of how emotional experiences like experiencing awe and the sublime unfold in an unmanipulated wilderness adventure context could bring forward knowledge of conceptual aspects of these experiences. This study aimed to investigate emotionally strong experiences from a bottom-up perspective of Arctic wilderness adventure and further contribute to the concept development of awe and the sublime in the interconnection between psychology and philosophy.

Our research question is as follows: How can narratives of Arctic wilderness adventure inform about awe experiences and the sublime, and what are the implications for understanding spiritual well-being? To answer this question, a qualitative approach was undertaken to collect bottom-up responses on how the informants’ expressions of powerful and meaningful Arctic wilderness experiences are articulated. A basic qualitative investigation was chosen to be able to make an inductive analysis of aesthetic nature experiences, where we were interested in how people interpret their experiences, how they construct their worlds, and what meaning they attribute to their experiences (Merriam and Tisdell, 2016, p. 24). As compared to quantitative approaches, the richness of narratives could be helpful in understanding awe and the sublime in the meaning-making process of being in the wilderness.

The empirical findings contribute to addressing our philosophical question on the relationship between awe and the sublime, based on selected sources of environmental aesthetics and closely related fields (such as Berleant, 1998; Carlson, 1998, 2000; Stecker, 2005; Bergmann, 2011). Finally, these findings are discussed in framing spiritual well-being as a concept informed by psychology and philosophy.

## Materials and methods

With an interdisciplinary approach (Moran, 2002), empirical and theoretical approaches contribute to spiritual well-being in an abductive process. These contributions inform each other and are discussed through transdisciplinary integration, informed by empirical and theoretic perspectives. Such transdisciplinary insight strives to generate a more comprehensive understanding with transformative potentiality.

### Arctic wilderness experiences

To investigate spiritual well-being and awe experiences empirically, we explored narratives collected from nature guide students after an introduction to glacier guide course. This was an 8-day excursion or adventure, situated on Svalbard. The location chosen for this course was completely off-grid. Transported by boat, the group was left on its own with only equipment that was brought for this purpose. Participants slept in tents. Safety matters were highlighted, as polar bears live in the area. The course was organized with daily hikes from the campsite, most of the days on the glacier, which was an hour of hiking without trails. The landscape was rocky and desert-like in most places, but there were some Arctic plants, particularly where there were bird colonies. There was no facilitation for hikers in the area, making, for example, river crossings challenging. It was in August, and the weather shifted from snow and rain to sun.

### Sample

Altogether, 27 participants out of 34 (a response rate of 76%; 16 men, 9 women, and 2 did not give gendered information) in a group of nature guide students agreed to participate in the research project and gave two distinct narratives of their Arctic wilderness experiences. This provided a total of 54 narratives for analysis and interpretation. There was a mix of Norwegian students and international students, with slightly more international students represented in the group. In addition, three male teachers and one female teacher assistant followed the group through 8 days of guide training in the glacial landscape. A particular focus was on safe techniques for responsible guiding in glacier areas, including guiding on blue ice. Rescue techniques were taught. The course also included elements of Arctic wilderness, geology, botany, cultural history, and Arctic zoology. The students were housed in large, military tents, with separate cooking tents, which needed safety procedures for polar bear protection, including odor control in the food and toilet structures and polar bear traps with explosives around the campsite. The course was part of the standard training program for Arctic nature guide students. This was the first outdoor course in the first semester of Arctic nature guiding, in which Arctic camp experiences were new to most of the informants. The location for the course was chosen to combine a safe camp with access to drinking water within a short distance of a glacier that was easy to access. Moreover, the location had to be approved by the local government for likeliness of avoiding polar bear attacks. All students and teachers were trained in self-defense in advance in case of a polar bear attack.

### Procedure

Inspired by the event reconstruction method (Grube et al., 2008), the group of Arctic nature guide students was asked to share their experiences using pen and pencil immediately after the course on Svalbard. Their narratives were informed by two questions: (1) “Please recall a situation where you felt interested. Please describe in your own words where you were, what you were doing, your sense experiences, or other descriptions of the experience.” (2) “When thinking about the whole trip, what do you associate with a strong experience of nature? Select one moment of this association. Please describe this moment.” These questions were informed by the discussion in psychology on the closeness of the feelings of “interest” and “awe” but also on our previous findings that aesthetic experiences ask for a “strong experience of nature” (Løvoll et al., 2020). Narratives that were responses to the first question were coded “a” and the informant’s number, and narratives that were responses to the second question were coded “b” and the informant’s number. Informants were numbered in random order.

### Analysis

The length of the narratives was from 13 to 252 words; most were about 100 words. Inspired by reflective thematic analysis (Braun and Clarke, 2006, 2021), the whole material was interpreted. First, the researchers familiarized themselves with the data by reading the narratives several times. Second, the researchers agreed to interpret the material together. Although this is not necessary for thematic analysis, we argue that this is the best way to analyze the material in an interdisciplinary manner. Thus, the codes and subthemes were all a part of an abductive interpretation process.

Third, through the coding process, 183 distinct codes were identified and agreed upon. Many of the codes were commonly used; for example, “vastness” appeared 9 times, “wonder” appeared 11 times, and “learning” appeared 10 times.

For example, the following narrative generated eight codes:

Since the camp was out in the wild, I expected to feel some connection with nature, but busy schedule kind of affected the experience, besides night polar watch, I did not find much time to observe/enjoy pure nature outside of setting camp in the nature. BUT I enjoyed my polar bear watch (3–5 AM both times). The rising sun over glacier, giving light on the mountains and silence made me feel privileged to stay there, be there and feel so small but part of the nature around me. I choose to have a second polar bear watch for the same time for that reason. (Female, 3b)

The codes generated from the quote above were “expected to feel connection with nature,” “solitude,” “vastness,” “part of the nature,” “feel so small,” “connection,” “polar bear watch,” “pay attention/awareness,” and “night (3–5 AM).” Fourth, 18 subthemes were found to sort the main findings from the material. During the abductive process of organizing subthemes and main themes, three main themes were identified and developed through the refinement of the original themes.

### Ethics

Participants in the research project gave voluntary consent by the agreement to deliver their narratives for the research project, and they were informed that they could withdraw without providing an explanation. The delivery of their text had no relation to their assessments or any other influences on their study. Information about the project and the researchers’ contact information were given. No personally identifiable information was collected. All collected data were paper-based. The data were fully transcribed and stored safely using OneDrive, which is safe for storing data. During the 8-day experience, the first author had the role of assistant teacher. She was not in charge of the course content or communication before, during, or after the course. She assisted in some technical skill training (training for rescue from crevasses) and socialized with a group of 10 students. It can be criticized that the presence of a researcher could have influenced the results, but these roles were not in conflict. The informants were invited to participate in the research immediately *after* the experience, and the material was collected by others, not the researcher. The presence of the researcher during the experience might have inspired participants to be aware of emotional experiences, but what content the researcher was looking for was not evident, other than describing interesting and powerful experiences during the adventure.

## Results

Narratives from the 8-day expedition covered many aspects of awe as having strong emotional experiences. On one of the last days of the stay, there was a polar bear swimming across the shore close to the campsite. All informants were able to see it from a safe distance. Eleven informants mentioned this episode as one of their strongest experiences. Among these, five informants reported this as an interesting experience, and six informants reported this as a strong experience of nature. However, the remaining twenty-five participants selected other episodes for their narratives. Five informants described their strongest moment during their polar bear watch at night, without seeing a polar bear. In this situation, they were alone, and it was silent and summer twilight. Furthermore, episodes on the glacier, around the campfire, or from hiking in the area, where both interesting episodes and strong experiences of nature took place, were all mentioned.

**FIGURE 1:**
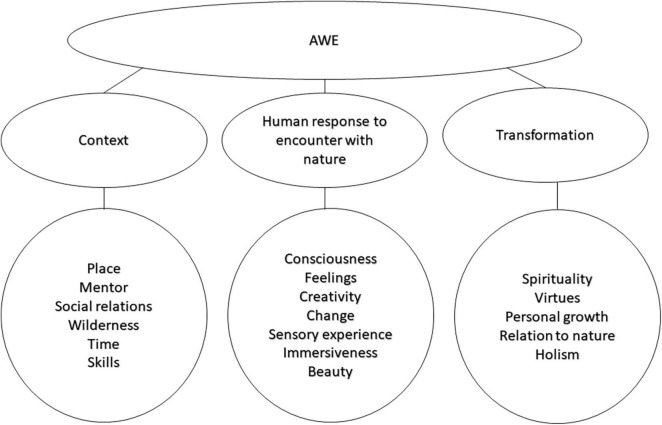
Main themes and subthemes of awe from Arctic experiences.

Three main themes of Arctic awe-inspiring experiences were identified: (1) *context*, with the subthemes place, mentor, social relations, skills, wilderness, and time; (2) *human responses to encounters with nature*, with the subthemes consciousness, feelings, creativity, change, sensory experience, immersion, and beauty; and (3) *transformation*, with the subthemes spirituality, virtues, personal growth, relation to nature, and holism (see Figure 1).

### Context

The context of the awe experience points to the dimensions necessary for these experiences to happen. The six subthemes, place, mentor, social relations, wilderness, time, and skills, were summarized from the narratives (subthemes in italics).

First, the qualities of *place* refer to the wilderness experience in many ways. Descriptively, it is about fragile vegetation, the presence of animals and birds, cultural landscapes, exposure to rocks and ice, and the shifting daylight. Some informants reported that there were many situations during their stay in the wilderness that promoted these powerful experiences. In particular, the polar bear watches at night, when informants were watching the campsite alone, were mentioned as important situations. Moreover, some situations during the adventure were more frequently mentioned than others, for example, when walking on the glacier for the first time, sitting around the campfire in the evening, and watching ice calving into the lagune. Attraction to place was also mentioned in relation to feelings of belonging and feeling affiliated with the place as a conscious reflection.

The context was also related to the special condition of *social relations*, with the existence of teachers (one teacher for a group of 10 to 12 persons). One group also had an assistant teacher. These teachers acted as *mentors* for various aspects, such as facilitating experiential learning, storytelling, and general awareness of the Arctic wilderness. The mentors were also role models for the nature guides in developing awareness of place and context. Being in a core group of 10 to 12 persons is a social experience. Many informants highlighted the social dimension of the adventure, in sharing thoughts and developing friendships, as well as working together and communicating in complex cooperation situations. The complexity of understanding place, social relations, and mentors, is expressed by one of the informants:

I felt interested throughout the whole trip, interested in the surroundings, the glacier, the course, my new fellow students, my own feelings during the excursion and how it would be to be out on Svalbard, staying out, with the weather and the polar bear situation. […] Maybe the strongest interest or wondering was on the first day, to set foot on the glacier to be in the unknown and to be “there”. Depending on each other, the guide and yourself while knowing we all feel excited and maybe “a bit” intimidated by the nature as well. I think it is special to feel how fast you learn to be there and learn the skills and also have time to look around and be on Svalbard. (Female, 14a)

When the students practiced rescue training in the crevasses, these operations were also challenging. Part of the program involved training *skills* to move on ice but also to cope with rescues as expertise. Effective technical skills were trained, such as building a 6:1 pulley system with the rope.

The *wilderness* experience included rich encounters with wildlife, such as polar foxes, birds, and seals, and even an unplanned encounter with a polar bear that came swimming along the campsite in his hunt for seals. This provided an opportunity to study polar bear behavior without approaching it (which is forbidden by law); when the students observed the polar bear swimming, they did not know whether it was an “authentic” polar bear hunting seals or a “man” polar bear looking for human food supplies. Fortunately, it was a healthy “authentic” polar bear. The observation that the polar bear failed to hunt seals from icebergs made a strong impression on several informants. Moreover, the wilderness experience related to the mindset of being present includes qualities of *time* perception. Some informants mentioned the need to stay in the wilderness for a length of time, which influences mindset. Others mentioned relaxing moments, the time to develop closeness to nature, and the quality of seeing the adventure as a whole.

### Human responses to encounters with nature

The context triggered many human responses in encounters with nature. The next main theme was the most comprehensive theme and consists of seven subthemes: consciousness, feelings, creativity, change, sensory experience, immersiveness, and beauty.

The subtheme *consciousness* was identified as attention and awareness to sensory experiences, attention to scenery or details, positive expectations, savoring memories, and aspects of fulfillment in reaching these places. Sensory experiences include smelling, seeing, and feeling (tactile experiences), experiencing light, odors, and the sound of ice, and being wet and cold. The night experience with a polar bear watch was often mentioned along with experiences of quietness, solitude, and silence. Experiences in the Arctic also contained a wide range of *emotions*, including negative and positive emotions. Feeling amazed was related to learning and being frightened. Feeling scared, excited, or surprised also seemed to be typical. Hedonic feelings, such as pleasure, happiness, love, and enjoyment, appear in the narratives, as well as negative emotions such as fear, feeling adrenaline in the body, being uncomfortable, and being frightened. Complex emotional experiences also included humbleness and expressions of mixed feelings, such as feeling curious, frightened, excited, and shaky at the same time.

One informant expressed her emotional experiences like this:

For me, the first day and first steps on the glacier was something special. I felt that was extremely interesting because we could finally use the equipment and practice what we have read about. It was specially interesting for me because it was different in real than what I had in mind. Everything was new, every was quite different than I expected. Learning how to walk with the crampons, using the ice axe, learning about the terrain, having the lunch on the ice. It was like accessing to a brand new world. And I was giving myself 100% to it. Of course, the next days have been also very interesting. Because I felt that the more, I learned, the more I was into it and eager to learn even more. (Female,15a)

There is also a *creative process* identified in aspects of discovery, novelty, and perception of new formations and new colors. The context was especially rich in the development of imagination as a response to the experience. Imagination, which was a frequent code in the material (seven times), is new individual experiences as well as a result of storytelling and increased awareness of place. Changes were mentioned as strong affective moments: changes in light, weather, and scenery.

Moments selected for the narrative are often referred to *immersion*. Words such as “magic moments” (Female, 15b), “thrilling” (Female, 15b), “loose breath” (Female, 4b), and “being moved” (Male, 19a) indicate immersion in the experience. In addition, characteristics of flow experiences, such as “transformation of time” (Male, 19a), are mentioned. Experiences of *beauty* were characterized by experiences of color descriptions, such as “vegetation in a strong green tone” (Male, 23a), variation, overview, harmony, scenery, uniqueness, landscapes, and contrasts in detailed descriptions. “Waves in the water” (Male, 6a) and “huge waves” (Male, 24b) were some direct descriptions of beauty.

### Transformation

Another main theme was transformation. This theme consisted of five subthemes: spirituality, virtues, personal growth, relation to nature, and holism.

Typically, these quotations were expressions of *spirituality*, such as “to be in nature give life meaning.” Other aspects relating to spirituality refer to vertical height difference, standing on the top of ice or a mountain, an experience of space, and direct expressions of awe: “To see an animal hunt like that, and be inside the perimeter of that hunt filled me with awe” (Male, 7b). Some informants felt a deep connection to the polar bear. Expressions of vastness, wonder, and power were all very frequently reported codes as expressions of spirituality. Wonder was most often reported (12 times), followed by vastness (9 times) and power (6 times). Specifically, experiences of belonging, awe, and self-transcendence were mentioned. An informant provided an example of wonder:

[…] coming across remains of humans living by the coast at Svalbard, it filled me with thoughts. Leaving the civilization to go north hunting, with barely nothing […]. What motivations can one possibly have for doing such a thing? Did they find the same solitude and peace in the nature as we do? With all these questions in my hand, the nature around me, grew into a much wider and stronger environment that made me reflect in what kind of relations we humans have had to nature in the past, and how we have grown from fighting against nature, to how we now fight for the nature. (Male,7a)

The subtheme *relation to nature* identified reflections and attitudes such as feeling like a guest in nature (in the world of the polar bear), expressing nature as superior, but also feeling connection and closeness to nature. Many expressed their feeling of being small in nature, while others felt part of nature. Moreover, affiliation with nature was expressed, despite being frightened, and being part of life cycles and the ecosystem. Feeling energized in nature was also mentioned.

One of the largest subthemes was *personal growth*. Typically, an eagerness to learn and learning from experience were highlighted. General learning was frequently mentioned (11 times). This could be related to many aspects, from learning skills to reflection, understanding more of the Arctic nature, self-knowledge, and getting new insights (five times). Personal mastery experiences were also commonly reported. Other dimensions relevant for personal growth were related to perceptions of challenge, acceptance, revelation, respect, recognition, realization, and motivational triggers. Increased feelings of responsibility were also part of the increase in understanding of the guide role. Observing the polar bear behavior triggered reflection. A fourth subtheme, *virtues*, appeared as aspects pointing to moral responses. Most of all, empathy was presented (seven times). An example of empathy appeared:

[…] when we were having a break, two Arctic foxes came pretty close as well, obviously curious, but not too scared. This for me was a very strong experience of nature, where the animals did not see us as enemies, but as a part of the environment surroundings. (Female, 10b)

Moreover, expressions of gratitude, appreciation, and environmental concern were mentioned. The seriousness and experiences of valuable elements were identified. In addition, a philosophic attitude of questioning and curiosity was mentioned.

The last subtheme, *holism*, the interaction of many elements, feeling part of the ecosystem, and the expectation of feeling connected to nature, was typical. One informant even expressed a grand narrative about nature:

We could see the evolution in front of our eyes, at scale. How the big mountains were transformed into peaks and later into grovel and sand by glaciers and other elements. How life alternated between birth and death, and how such an introspectable dee allowed for so much life: plants, animals that exploited every single resource. It was something to discover connections and relations among different parts of nature. (Male, 12b)

This quote also reflects on birth and death as a part of the life cycle.

### Intertwined connection between themes

In the exploration of how awe can be characterized, the three main themes cover aspects of the experience. In three quotes, the intertwined connection between themes and subthemes becomes evident. One of the informants expresses his/her transformative experience during an episode of being aware of rocks:

A strong experience of nature is for me when one understands, or starts to understand nature in which one stands. Understanding how the rocks fall, how long, how old are they, the crevasses, the behavior of polar bear and foxes. Understanding brings closeness. Pete spoke about the age of the rocks and explained: If you walk a long that ridge, you travel from one meter to another the more than 200 000 years in time. This is unbelievable. Without this information, it would have been just rocks. (Female, 2b)

The *context* relates not only to the wilderness experience, with specific references to animals, rocks, and glacier crevasses, but also to the presence of a mentor (Pete). Moreover, imagination inspired by the history of rocks is an expression of creativity and is a *human response to an encounter in nature*. Wonder at the perception of how rocks fall and the geological development trigger spirituality and personal growth in getting new insights cognitively as well as emotionally in the statement “this is unbelievable. Without this information, it would have been just rocks.” This points to *transformation* through spirituality, insight, and personal growth. Another informant expresses his/her transformative experience during an observation of the polar bear:

I think my strongest sense of nature was realized when I was most uncomfortable and cold, tired, and soaked to the bone. During these moments I gained a large amount of respect for the wildlife that deals with these forces of nature and struggles and survives every day. I felt empathy for the polar that swam so far and failed to catch a seal. I could almost see his frustration as he stood on the iceberg and then laid down to rest after his failed attempt. I felt bad, as I was able to eat my soup and food comfortably as he sat on the iceberg sullen and dismissed. (Male, 5b)

In this quote, the *context* informs us about a situation that is highly unpleasant. There has been some rain and exhausting elements. In addition, there is rich wildlife and ice, sea, and an encounter with a polar bear. The informant is wet, cold, and tired. Despite these negative feelings as a *human response to an encounter in nature*, the informant explains a vivid imagining of how the polar bear dealt with his harsh environment. This is further explained as a *transformative* process through wonder, development of virtue (empathy), and even a strong identification with the polar bear that failed to kill its prey. A third informant expresses his/her transformative experience by paying attention to the glacier calving:

A strong experience of nature for me was standing up to the glacier watching a giant (ice) calving down in the lagune and feeling the whole glacier shaking. Then the calving made a huge wave to that moved towers of ice. (Male, 24b)

In the third quote, the *context* is being close to the glacier, watching a giant ice calving into the lagune. This experience is articulated as a sensory experience of the whole glacier shaking, as a *human response to an encounter in nature*. Attention is then directed to the huge wave, which moved towers of ice, indicating the immense power of nature, and being moved by this event. This points to *transformation* in the sense of sublime experiences of beauty.

## Discussion

Based on the identification of awe-inspiring experiences in the Arctic wilderness, the themes *context*, *human responses to encounters with nature*, and the *transformation* appear as necessary, typical, and commonly reported aspects of the total experience. Across the material, these aspects were often combined in the narratives. The data collection provides a rich approach to understanding the complexity and the spiritual power of wilderness experiences (see Figure 1). As a general interpretation, the narratives inform about commonly experienced characteristics of awe and the sublime from the perspective of associative text reflections after returning to civilization. This new way of collecting data on awe-inspiring experiences provides insights into the further exploration of this phenomenon, where the two questions that informed the narratives (interesting and strong experiences) had overlapping, yet different, contributions to the articulation of experiences.

The three main themes of Arctic awe-inspiring experiences give a direction for how to theoretically explore such experiences. To shed light on this, we carefully move from the empirical context to an integrated understanding of the awe-inspiring experiences, drawing on resources from within an interdisciplinary framework. Our findings and the construct of three themes echo in the interdisciplinary areas of philosophy, psychology, aesthetics, and spirituality. The resources from these fields are all related to the interaction between humans and nature, such as environmental philosophy, philosophical aesthetics (in particular, the “turn to nature” within this field), and nature spirituality. This integration is our point of departure for the theoretical discussion. However, some of the subthemes can—in one sense—be related to theoretical discussion (such as spirituality). Together, the main themes provide resources for the notion of spiritual well-being.

### The need to understand the context

It is nature *as context* that lays the foundation for the whole scene for exploring awe. Thus, descriptions of nature and wilderness as places seem quite self-evident. Some of the preconditions for context are identified as places in a broader sense, for example, wilderness and social relations (being together and the role of mentoring), but also more specific places and phenomena (e.g., glaciers and animals present in a specific environment). One overlapping aspect of the relationship between context and human response to the encounter with nature is *awareness of one’s surroundings*. Our findings have nature as an assumptive context and a formative one. This can be explored by considering the notions of space and place.

One starting point is Henri Lefebvre (1991) triadic approach to the spatial turn (which was later addressed in a wide range of disciplines) that emphasizes physical, mental, and social spaces as *spatial practices*. Peter Nynäs (2008) followed up Lefebvre’s space by addressing the slightly overlapping concept of place. For Nynäs, place has three dimensions: one physical-geographic, one sense-emotional, and one socio-cultural. By highlighting these notions, we have resources for a broader and deeper understanding of humans’ interaction with nature, with high relevance for our findings.

In addition, we can understand space and place as dynamic concepts (Aure et al., 2015, p. 13ff). Places are created, and they have a dynamic character in the interaction between human and nature, in Arne Næss (2005) terms an “internal relation” (p. 339). Such an internal relation between humans and nature can be understood as intertwined in terms of internal space (humans) and external (nature, our surroundings). Sigurd Bergmann sees them as belonging to a common continuum and being part of a deeper reality, which grounds, anchors, and embraces the living. Such a continuum opens to seeing nature as the context for inhabitation and as home (Bergmann, 2011).

Furthermore, the experience of being a part of nature does not necessarily take place in solitude. We find social relations (between students and between a mentor and students) an essential element. Thus, the community of shared experiences needs to be emphasized. An important part is that the role of the mentor is related to skills. Skills in this context must be understood broadly. Skills span glacier hiking techniques, rescue techniques, how to pay attention to one’s surroundings, and the learned skill of openness to nature. Furthermore, awareness—often addressed as “to pay attention”—is about *time*. We can identify time as being necessary for developing an awareness of one’s surroundings and skills (expressed in our findings as the length of the stay), having relaxing moments, developing closeness to nature, and seeing the adventure as a whole.

The wilderness adventure, with all its characteristics of organizing, planning for learning situations, socializing in a group, learning new skills, the role of mentoring, living off-grid by and with simple means for more than a week, and in a certain place with rich natural attractions, thus, is a necessary part of understanding how awe experiences emerged. With other framings, the output of the adventure would be different.

### The thin sublime experience: Human responses to encounters with nature

The largest group of subthemes was human responses to encounters with nature. This theme was very much informed by the perspective of “here and now,” containing feelings and sensory experiences, including positive and negative valences. However, mixed feelings could also be expressed, such as curious, frightened, excited, and shaky. Tactile experiences, shifting daylight, odors, sounds of ice, but also silence, and being wet and cold are examples of sensory experiences. Mixed valence is a typical ingredient of overlapping elements between awe and the sublime (Clewis, 2021). Paying attention involves all of our senses: what we see, hear, taste, and touch. For Bergmann and Eaton (2011), such an openness “influences the kind of questions we ask, how and what we reflect upon and ultimately how we answer our queries” (p. 3). Thus, there is a line between how we experience the situation and how we reflect on it, and how this reflection meets our search for answers. This line of thought is also supported by the communicative theory of emotion (Oatley, 1992).

As a theoretical interpretation, sensory and emotion states typically refer to the description of “thin sublime” (Shapsay, 2019). However, several other subthemes can also follow this immediate affective arousal, such as attending to details, being totally immersed, or suddenly being aware of new sights, thoughts, or changes, which could start a creative process. Experiences of beauty could moreover support thin sublime experiences, including awareness of color descriptions, overview, scenery, uniqueness, variation, and contrasts.

As experiences of vastness and accommodation were the typical characteristics of the pioneering work of psychological identification of awe experiences and the sublime (Keltner and Haidt, 2003), this theme seems to fit well with the original psychology of awe experiences. Perceived vastness could be related to aspects of beauty, such as overview and large scenery, while the need for accommodation can relate to many of the surprising or new elements, elevating attention to objects, feelings, thoughts, and the aesthetic experience of nature. The factors captured in the Awe Experience Scale (Yaden et al., 2019) expand this theme with connectedness as one of the factors we identified as “transformation.” The other factors of altered time perception (self-diminishment, connectedness, physical sensations, and the need for accommodation) fit well with our inductive findings within this theme. In addition, aspects of consciousness, such as “positive expectations,” “savoring,” “paying attention to details,” and “awareness,” appeared in the data material. Aspects of creativity, such as “discovery,” “novelty,” and “imagination,” could be a part of the accommodation, but point to what these accommodation processes direct to. In the material, experiences in the wilderness influence how the informants think about themselves, others, and nature. In particular, “imagination” was frequently mentioned.

Overall, the subthemes have aspects of a positive or meaningful experience, which often have a self-reflective and moral character (Schlegel et al., 2016; Steger, 2016). For these experiences, mixed emotional valences make sense. Pure hedonic experiences, such as pleasant sensory experiences, feeling comfortable, or experiencing beauty as positive feelings, can also be part of what we can interpret as “thin sublime.” Thin sublime experiences thus include a richness of ways we can feel immediate affective arousal and be inspired by these moments. To sum up, our empirical analysis adds some nuances to the richness of understanding “thin sublime” experiences.

The theme *human responses to encounters with nature* points to psychology and fits well with the empirical study of awe experiences as it is studied in quantitative studies (Yaden et al., 2019). However, subthemes like immersion, creativity, and beauty expand the descriptive study of awe and need further philosophical investigations.

### The thick sublime experience: Transformation

The third theme, “transformation,” covers subthemes that expand the descriptive study of awe. Here, a change toward new ways of being self-aware and aware of developing values takes place in the subthemes of spirituality, virtues, personal growth, relation to nature, and holism. The findings add empiric knowledge to the value of wilderness experiences for the development of spiritual well-being (Ryff, 2021). These findings also make sense to earlier research on aesthetic nature experience, where a two-factor model suggested a difference between beauty and the sublime (Graves et al., 2020). Building on these findings, understanding the sublime part of awe experience seems to be essential for grasping experiences in nature, distinct from the traditional appreciation of beauty, and needs further investigation.

By addressing transformation as rooted in thick sublime experiences, we acknowledge sublime aesthetic experiences as complex and with a need for philosophical investigations. Transformation is empirically identified as psychological well-being (personal growth), moral awareness (virtues), and spirituality (holism and relation to nature). Spirituality separately appeared as a large subtheme that also contained wonder, vastness, and power as frequently mentioned topics, among other characteristics. How spirituality can be related to well-being is complex. Washington (2019) understands spirituality as an attempt to place and maintain ourselves in a healthy or correct relationship with the ultimate source of value, meaning, and perhaps life itself. He states the relationship more clearly with a reference to environmental ethics by saying that a core element of environmental ethics is to restore the broken relationship between humans and nature. According to Washington (2019), this restoring—or even healing—process is of major importance, because our mental, physical, and spiritual health depend on it.

According to Fuller (2012), the notion of wonder can be helpful for understanding transformation in terms of *virtues* and *spirituality*. For him, one way of understanding the connection between aesthetic experience and virtues is biological, in the sense that evolution favored a specific motivation program identified as moral emotions (Keltner and Haidt, 2003; Fuller, 2012). For Fuller, humans have a biological-based capacity to respond to vastness in a way that expands our possibilities of thought actions (Fuller, 2012). In particular, the expanding element can be identified as the feeling of being a part of a larger whole. Of interest here is Fuller’s perspective on these thought actions: They support human abstract thinking. New experiences—such as awe in nature—“disturb” what he describes as the established balance between human cognitive structures and the surrounding world in a way that supports cognitive development. What fuels these thought actions is wonder, according to Fuller. Wonder draws us toward what we wonder about: “Wonder motivates a quest for increased connection with the presumed source of unexpected displays of life, beauty, or truth” (Fuller, 2012, p. 78). We identify this cognitive development within the subtheme *personal growth*, in terms of gaining new insight, developing new skill support, and learning. The expanding character of wonder—evoked by awe—initiates even further contemplation than “just” wondering about what is observable. Such contemplation can be identified as spiritual. For Fuller, wonder stimulates contemplation of what is behind observable phenomena. This “behind” is not identified as such by Fuller, but addresses a direction, toward “cosmic issues,” which concerns the topics of meaning, purpose, and existence. Spirituality is moreover nourished by wonder (Washington, 2019, p. 182). Wonder—also a core element elicited by aesthetic experiences in nature—brings forth a response that has a (trans)formative character (Løvoll et al., 2020). Transformation is about activating specific virtues, stimulating personal growth, and providing a deeper understanding of humans’ relationship with nature.

How *relation to nature* yields transformation is complex. The experiences of being connected and close to nature and of experiencing nature as superior and only being guests in nature illustrate this complexity. These paradoxical feelings—being a guest and feeling at home—echo how sublime and awe experiences in nature are often identified (Bethelmy and Corraliza, 2019; Clewis, 2021) as having positive and negative elements. We feel attraction, yet might feel a need to withdraw. We experience belonging and at the same time alienation. The strong intertwined connection between the experience of power, vastness, connection, and closeness can be identified among the students in different contexts, as well as in their encounters with animals.

An important part of the transformative character of awe experiences is to recognize aesthetic experiences (i.e., sublime experiences in nature) as having an intrinsic value and therefore related to *virtues* and ethics. Fuller (2012) describes that awe experiences in nature have the potential to transform us toward a reorientation of our goals and values. Thus, such experiences have transformative potential with a future direction: what we hope for and what we aim and strive for. The intertwined relation between aesthetic experiences and ethics is also described by Bergmann with the term aesth/ethics (Bergmann, 2011). This notion highlights that ethics is continuously embedded in perception. Bergmann’s notion of aesth/ethics helps us to clearly understand humans’ responses to encounters with nature (where aesthetic experience is an important part) as closely related to values (i.e., ethical considerations). Transformation, in terms of fostering *virtues*, is evident in our findings; one of these virtues is empathy. Thus, awe experiences foster empathy and initiate moral responses.

In the subtheme “holism,” the interaction of many elements and feeling part of the ecosystem became evident in the narratives. Holism relates to the theory that parts of a whole are in intimate interconnection, and in the field of spirituality, holism is identified as a goal for meditation or contemplation (Washington, 2019). Holism is then about a transformation of oneself by gaining new insight into how the whole nature (reality) is deeply intertwined, such as our finding about experiencing a grand narrative about nature. Such a holistic experience is closely related to self-transcendence. Self-transcendence, as a realization that we are one small part of a greater whole, can be understood in several ways. That which is a greater whole can be nature, the universe, but also something beyond the visible (e.g., divine power). Therefore, holism is not only about grasping the interconnectedness of the visible in nature but also acknowledging that awe experiences are self-transcendent (e.g., make room for spiritual ideas).

### On the concept of awe and the sublime

Based on our empirical findings, awe experiences are typically experienced from the 8-day expedition. They carry elements from both the thin and thick sublime experiences, as they both are affective and transformational, and they take place in a certain context of nature, where social relations, the role of the mentor, learning new skills, and several other characteristics are important to make these experiences happen. Our second theme, human responses to encounters with nature, can be supported by the identification of “inspiring energy” in the factorial analysis of Bethelmy and Corraliza (2019). This also resonates with the two-factor model of beauty and the sublime, as distinct factors (Graves et al., 2020). In the strive to find content to the conceptual understanding, it gives sense to identify one aspect of awe and the sublime as being more affective than the other. Moreover, the affective approach can be associated with the “thin sublime,” which is the powerful affective experience (Shapshay, 2019), as well as the attention directed to aspects of the wilderness experience that inspire us.

Although the study of affective processes is highly psychological-oriented, it is necessary for the ability to develop aspects of the thick sublime, typically informed by highly intellectual aesthetic responses, such as wonder, virtues, relation to nature, and holism. These experiences would be more typical and spiritual, according to the element of intellectual thinking as a response to emotional inspiration. Nevertheless, the context for these processes to happen must be considered, in which this study of Arctic nature guide students was a truly relevant option to collect naturally occurring awe and sublime experiences.

The theoretical interpretation of thin and thick sublime was made after the data analysis. However, these distinctions fit well with our empiric findings in theory development in the identification of the main themes as distinct aspects of the experience. The distinction between thin and thick sublime, presented by Shapshay (2019) and Clewis (2021), is inspired by Kantian philosophical aesthetics. In this thinking, the sublime relates to reason and cognition more than to emotions. Although this distinction between thin and thick sublime makes analytical sense, there are also arguments not to separate these aspects of the sublime. For example, from a neuropsychological point of view, emotional, cognitive, and motivational aspects are highly interconnected in the neuroplasticity of the brain (Crocker et al., 2013). Additionally, the main theme “context” points to many necessary aspects for making the thin and thick sublime experiences happen.

Emotions, moods, attention, and cognitive thinking are interwoven, and the causal direction for which factor pushes the other in human behavior is hard to identify. There could be arguments to see thin and thick, emotional, cognitive, and motivational aspects as interconnected. From the narratives, the identification of all three main themes within the same quotes about specific episodes points to the overall complexity in dividing the sublime into distinct levels of “thin” and “thick.” However, this distinction could help us identify the psychological and philosophical contributions to the theoretical development of awe and the sublime. In our analysis, both thin and thick sublime appear as ingredients in the sublime experience, but we have not examined a causal direction between them. Instead, the link between the psychology of awe and the philosophy of the sublime benefits from the pragmatic communication of terms and contents, informed by rich narratives as the empirical baseline.

### Environmental aesthetics: Toward an integrated understanding of awe, the sublime, spirituality, and well-being

By bringing forth a more holistic, nuanced, and complex understanding of awe, the sublime, and spirituality (developed in the three main themes), we can draw on selected resources from environmental aesthetics. This field spans a wide range of positions. Environmental aesthetics emphasizes the aesthetic experience and appreciation of nature, widely understood (Eaton, 2006). The term “environmental” indicates involvement with environmental issues. Therefore, environmental aesthetics addresses discussions of values and ethics in our encounters with nature. For Stecker, the relationship between what we appreciate and what we value is closely related. Stecker (2005) states succinctly that to value something presupposes an experience. Environmental aesthetics gives us theoretical resources for grasping how intertwined aesthetic experiences in nature are (i.e., identified as awe, the sublime, and spirituality). Consequently, this sheds light on the three main themes. Finally, environmental aesthetics provides a theoretical point of departure for further exploration of *action* in terms of environmental goals and practices.

We highlight a few selected aspects from Carlson (1998) models for understanding environmental aesthetics: the arousal, the mystical, and the metaphysical. The first, the arousal, pinpoints the emotional relation to nature. According to this model, two core approaches to understanding aesthetic experiences in nature concern emotional and imaginary responses. Such a view is advocated by Noël Carroll (1993). We find this approach evident in the theme of *human responses to encounters with nature*.

The mystical model emphasizes the experience of nature as a mystery. According to Carlson (2000), appreciation of nature involves a state of appreciative incomprehension. For him, such an approach can be partly associated with a religious approach. We identify the notion of mystery as closely related to spirituality. The notions of mystery and spirituality are not only related to religion in our research but also are understood as aesthetic experiences having a self-transcendent meaning. Thus, mystery can be identified in themes related to the transformative potential of awe experiences in nature.

The third model of interest is the metaphysical model. This can partly be related to the mystical model but articulates an even clearer self-transcending and transformative character of experiences in nature that are closely related to the imagination: “Our metaphysical imagination interprets nature as revealing things about the whole experience, about the meaning of life, about human condition, about humankind’s place in the cosmos” (Carlson, 1998). This quotation shows that those transformative experiences can include values and virtues and initiate holistic understanding.

Therefore, environmental aesthetics gives voice to a holistic, integrated, yet complex, understanding of nature. Holism can be understood differently in this context. One type of holism is informed by natural science: “We are all bound up in one great natural system, an ecosystem of universal proportions in which no part is immune from the events and changes in the others” (Berleant, 1998). However, holism can also be articulated by the complexity of the factors involved in the experience. According to Berleant (1998), the aesthetic experience of nature involves a wide range of factors, such as space, volume, time, movement, color, light, smell, touch, and meaning. These factors resonate in a unique way with context, human responses to encounters with nature, and transformation. By including a variety of elements in the aesthetic experience, environmental aesthetics shows the complexity of experiences in nature. These insights from environmental aesthetics do not exclude each other but can be seen as complementary for doing justice to the complexity of aesthetic experiences in nature (Budd, 2003; Stecker, 2005). In addition, the complexity displays the need for an interdisciplinary—holistic—approach, which is at the core of the agenda for environmental aesthetics.

By mapping out these models, which resonate with our findings, we argue for a nuanced and complex understanding of awe, the sublime, and spirituality, developed in our three themes. However, we also pinpoint the potential for action from these insights. With action in this context, we address how we can understand transformative aesthetic experiences in nature as promoting ethical concerns (Carlson, 1998) and spiritual well-being. Seel (1998) puts it as follows: “the aesthetic of nature is (…) simultaneously part of an ethics of the individual conduct of life (…) for aesthetics, being concerned with specific forms of and opportunities for process-oriented activity, is generally part of an ethics of the good life” (p. 342). Seel binds together the aesthetics of nature with ethics and well-being (in terms of a good life). This quotation also illustrates the agenda for environmental aesthetics as having environmental goals and initiating practices. We identify one of these as spiritual well-being.

To sum up, experiences in the wilderness are a valuable setting to study the close relationship between awe and the sublime and their implication for spiritual well-being. Our conclusion so far is that the concepts of awe and the sublime are equally important for understanding spiritual depth. However, they are distinct aspects of the spiritual experience. The affective aspect benefits from psychological investigations, while the transformational aspect exceeds the psychological construct of awe. Facets of the experience happen at the same time in human consciousness, yet needs different explanations (Searle, 1999). Affective experiences could have causal explanations, while spirituality could have intentional explanations, in which the qualitative approach contributes to research on meaning-making. Moreover, the *context* of identifying awe and the sublime must not be overlooked. This is also a core message from Nynäs (2008) in understanding how place influences human beings on at least three different experiential levels. An essential question thus addresses the role of context: Are conceptualization of awe and the sublime within measurement scales valid? If we eliminate the place and only identify mental representations of awe and the sublime, we give intrapsychic explanations to complex phenomena, which also need ecological explanations. To overcome these challenges, a new ecological framework of spiritual well-being needs to be developed.

### Implications

The qualitative exploration of awe and the sublime in relation to how we understand spiritual well-being points to several implications. First, the qualitative empirical investigation found awe and sublime experiences as typically reported for an Arctic adventure. The wilderness setting of pristine nature, with the presence of wild animals and being away for 8 days, offered a good framework for studying awe, the sublime, and spiritual well-being.

Second, the bottom-up approach, by analyzing text quotes informed by the two questions, added a rich and nuanced understanding of the intimate relationship between awe and the sublime, which is essential for the further development of measurement scales. What phenomenon is going to be measured needs careful investigation from the intersection between psychology and philosophy. The progress in this work is promising, but more studies are needed to develop valid instruments.

Third, the close relationship between spirituality and eudaimonic well-being was typically identified in the text quotes relating to the identification of positive and powerful experiences. Many aspects of eudaimonic well-being, such as connectedness, learning, creativity, being immersed, and being self-aware, were directly identified in the material. Similarly, for spirituality, as virtue development, wonder, relationship to nature, and holism are typical aspects of the experiences reported from the adventure. Consequently, the importance of wilderness adventure with longer stays with simple living needs to be considered for larger groups in society, promoting well-being and awareness of nature in a world where pristine nature is a threatened natural resource.

Fourth, the theoretical development of the psychology of the sublime benefits from the environmental aesthetics field and, in particular, holistic approaches to seeing experiences in nature as powerful sources of the sublime. Bodily experiences, sense experiences, the place, the relation to nature, and other people can be seen as part of understanding the larger whole of human well-being, a view that might challenge the typical Western view.

### Limitations and further research

The empirical investigation of awe and sublime experiences, informed by philosophical insights, is still in an early stage of development. A series of empirical studies are needed within different contexts and samples of engagement with nature. The wilderness setting, several days of adventure, is promising for investigating awe and the sublime, and further research should investigate whether such themes and structures hold for adventures with less spectacular wilderness qualities, for example, on the mainland and various campsites. Longitudinal studies with a follow-up design after wilderness adventures would provide more knowledge on the importance of such experiences from a wider and long-lasting well-being and spirituality perspective.

The methods developed for this design, capturing narratives from informants, seem to be a valuable approach, but other methods could be used, such as daily diaries, interviews, and observations. In the sample, Norwegian and international students from European countries were represented. Thus, the phenomenon examined is not merely a Norwegian perspective. Moreover, study designs involving personal and cultural idiosyncrasies should provide more knowledge about how awe experiences in nature emerge dependent on such factors.

Taking the perspective of global environmental aesthetics, the holistic view of integrating spirituality and well-being could benefit from theories with non-Western origin. For example, there are conceptual understandings of the Japanese “Shinto,” seeing spirituality and well-being as a unity with seven characteristics: place, way, beauty, festival, technique, poetry, and ecological wisdom (Toji, 2016). Alternative theoretical frameworks to connect spirituality and well-being might put forward more complexity in our understanding of wilderness adventures.

## Conclusion

Our empirical findings and theoretical explorations bring forth transdisciplinary insights by providing a more profound understanding of experiences in nature, with the potential for transformation in our interaction with nature and well-being. Narratives from Arctic nature guide students after an 8-day glacier adventure course informed about rich aesthetic awe experiences and experiences of the sublime. The three main themes—“context,” “human responses to encounters with nature,” and “transformation”—provide insights into differences and similarities in the distinction between “thin” and “thick” sublime. Holistic interpretations are suggested in the framework of environmental aesthetics. The findings have implications for concept development and practices in promoting spiritual well-being.

## Data availability statement

The raw data supporting the conclusions of this article will be made available by the authors, without undue reservation.

## Ethics statement

Ethical review and approval was not required for the study on human participants in accordance with the local legislation and institutional requirements. The patients/participants provided their written informed consent to participate in this study.

## Author contributions

Both authors listed have made a substantial, direct, and intellectual contribution to the work, and approved it for publication.
